# Is There an Association Between Untreated Hearing Loss and Psychosocial Outcomes?

**DOI:** 10.3389/fnagi.2022.868673

**Published:** 2022-05-19

**Authors:** Dona M. P. Jayakody, Justin Wishart, Inge Stegeman, Robert Eikelboom, Thomas C. Moyle, Jessica M. Yiannos, Jack James Goodman-Simpson, Osvaldo P. Almeida

**Affiliations:** ^1^Ear Science Institute Australia, Subiaco, WA, Australia; ^2^Ear Sciences Centre, Medical School, University of Western Australia, Perth, WA, Australia; ^3^Centre for Health and Ageing, University of Western Australia, Perth, WA, Australia; ^4^Department of Mathematics and Statistics, Faculty of Science and Engineering, Macquarie University, Sydney, NSW, Australia; ^5^Department of Otorhinolaryngology, Head and Neck Surgery, University Medical Center Utrecht, Utrecht, Netherlands; ^6^Brain Center Rudolf Magnus, University Medical Center Utrecht, Utrecht, Netherlands; ^7^Department of Speech-Language Pathology and Audiology, Faculty of Humanities, University of Pretoria, Pretoria, South Africa; ^8^Department of Physics, Faculty of Engineering, Mathematics and Sciences, University of Western Australia, Perth, WA, Australia

**Keywords:** hearing loss, social loneliness, emotional loneliness, social support, social interaction

## Abstract

**Objective:**

Age-related hearing loss is one of the leading causes of disability in older adults. This cross-sectional study investigated the association between untreated hearing loss, social (perception of quality and quantity of social network) and emotional loneliness (perception of limited emotional support), social isolation (size of the social network), social support (actual or perceived availability of resources from the social network) and psychological discomfort (depression, anxiety, and stress) in older adults.

**Study Design:**

Cross-sectional study design.

**Methods:**

A total of 202 community derived sample of volunteers, age range 40–89 years, mean age (M) = 65.3 ± 11.0 years were recruited. Of these 115 were females (*M* = 63.2 ± 12.0 years) and 87 were males (*M* = 68.2 ± 8.9 years). All participants completed a hearing assessment, social interaction and support questionnaire and a social and emotional loneliness questionnaire.

**Results:**

Hearing loss significantly contributed to both moderate [*P* < 0.001, B (95% CI): 0.01 (0.99–1.02)] and intense levels [*P* < 0.001, 0.02 (1.00–1.04)] of emotional loneliness. Depression was significantly associated with satisfaction with social support [*P* < 0.001; −0.17 (−0.23 to −0.11), social interaction [*P* = 0.01; −0.07 (−0.12 to −0.01)], and moderate [*P* < 0.001; 0.31 (1.22–1.53)] and intense [*P* < 0.001; 0.29 (1.20–1.50)] levels of emotional loneliness and intense levels of social loneliness [*P* = 0.01; 0.12 (1.05–1.21)].

**Conclusion:**

Untreated hearing loss significantly increases the odds of being emotionally lonely. Depression significantly contributes to social and emotional loneliness, satisfaction with social support and social loneliness. Given the higher prevalence of loneliness and psychological discomfort and their associations with untreated hearing loss, hearing-impaired older adults are at significant risk of developing loneliness and psychological discomfort. Therefore, hearing health professionals should be aware of the psychosocial burden that may accompany hearing loss, in order to provide appropriate advice and support.

## Introduction

In 2019, 1 billion of the total world population was 60 years or older, which will increase to 2.1 billion by 2050 (World Health Organization [WHO], 2021a). As the number and proportion of adults aged 60 years and older are growing, factors related to these older adults’ health and wellbeing deserves attention. An individual’s social health depends on their capacity to establish meaningful relationships, adapt to social situations, and interact with social networks (Freak-Poli et al., 2021a). Therefore, we explore three distinct yet interconnected components of social health (Freak-Poli et al., 2021b): social isolation, loneliness, and social support.

### Social Isolation, Loneliness, and Social Support

Social isolation and loneliness are descriptive terms that are often used interchangeably, although they refer to two distinct concepts. Social isolation is an objective and quantifiable measure, while loneliness is a subjective emotional experience (De Jong-Gierveld and Kamphuls, 1985; Holt-Lunstad et al., 2015). Social isolation is characterized by a limited relational network and social contacts (Holt-Lunstad et al., 2015), while loneliness describes the unpleasant feeling resulting from the discrepancy between desired and existing social relationships (Pinquart and Sörensen, 2003; Boldy and Grenade, 2011). Loneliness can be further subdivided into “social loneliness” (the perceived lack of a good-quality social network) and “emotional loneliness” (the perceived lack of intimate/emotional support from a significant other) (Weiss, 1973; Gierveld et al., 2006). Thus, an individual’s perception of the size of their social network and the emotional connections they have will influence whether they feel lonely or not (Weiss, 1973; Gierveld et al., 2006; Cacioppo et al., 2015).

Even though the terms’ “social isolation” (negative) and “social support” (positive) are used interchangeably, they do not refer to diametrical concepts (Berkman et al., 2000; Holt-Lunstad et al., 2015). While social isolation is an objective measure of the number of social connections (Holt-Lunstad et al., 2015), social support is a subjective measure of the actual or perceived availability of psychological and material resources provided by the social network (Cohen, 2004). Social support further can be divided into three categories based on the resources: tangible (provision of materials), informational (guidance or support), and emotional (empathy, care) (House and Kahn, 1985).

### Impact of Social Isolation, Loneliness and Social Support on Health and Wellbeing of Older Adults

Loneliness is associated with depressive symptoms (Cacioppo et al., 2006, 2010; Zebhauser et al., 2014), reduced physical activity (Hawkley et al., 2009), decreased satisfaction with life (Zebhauser et al., 2014; Bai and Knapp, 2016) and poor subjective wellbeing (Pinquart and Sörensen, 2003). Loneliness has also been associated with cognitive impairment (Donovan et al., 2017; Kim et al., 2020), increased risk of dementia (Zhou et al., 2018) and cardiovascular disease, diabetes and migraine (Christiansen et al., 2016). Social isolation has been associated with falls, re-hospitalization, cardiac heart disease, cancer, and nutritional risk (Nicholson, 2012). Current literature highlights that loneliness and social isolation resulting from poor social relationships increase the mortality rate among older adults (Luo et al., 2012; Holt-Lunstad et al., 2015). On a positive note, social support increases the resilience to stressful events (Ozbay et al., 2007) and suicidal ideation (Zhang et al., 2018), plays a protective role against depression (Gariépy et al., 2016) and reduces the Diabetes burden (Kaya and Caydam, 2019). Identifying factors that contribute to developing strong positive social relationships in older adults could help reduce this population’s high morbidity and mortality.

### Hearing Loss and Its’ Consequences

Age-related hearing loss (ARHL) is prevalent in later life and has an increasing trend across ages; 10.9–17.6% in 60–69 years, 41.9–51.2% in 80–89 years, and 52.9–64.9% in 90 years and above (World Health Organization [WHO], 2021b). ARHL is associated with an increased risk of cognitive impairment (Jayakody et al., 2017), dementia (Dalton et al., 2003; Lin et al., 2011), and Alzheimer’s disease (Lin et al., 2011), poor quality of life (Dalton et al., 2003), physical inactivity (Gispen et al., 2014), social isolation (Strawbridge et al., 2000), as well as depression, anxiety, and stress (Jayakody et al., 2018). ARHL is characterized by loss of peripheral hearing sensitivity and decreased ability to understand speech, mainly when there is background noise (Davis et al., 2016). Thus, hearing impairment poses a major challenge as it impairs the ability to hear, listen and understand the intended message. Hence, the consequences of hearing impairment affect not only the hearing-impaired individual but their communication partners/significant others as well (Kamil and Lin, 2015). Communication partners experience poorer quality of life and relationship satisfaction, a restricted social life and increased communication burden, and (Kamil and Lin, 2015).

Communication refers to the “bidirectional transfer of information, meaning, and intent between two or more individuals” (Kiessling et al., 2003). Communication forms part of a social act which involves expressing oneself and relating to others and is moderated by the emotions, attitudes and beliefs of the communication partners and the rules of the society (Lemke and Scherpiet, 2015). Verbal communication requires both participants to hear, listen and comprehend (Kiessling et al., 2003). According to the World Health Organization’s International Classification of Functioning, Disability, and Health (World Health Organization [WHO], 2001), communication disability due to hearing impairment is an outcome of interactions between sensory impairment and participation in life. Hence, communication impairment resulting from hearing loss restricts active involvement in social and cultural activities leading to withdrawal and feelings of loneliness (Strawbridge et al., 2000) and social isolation (Pronk et al., 2011; Zebhauser et al., 2014). Several studies have found that hearing loss is associated with a higher risk of social isolation and loneliness (Pronk et al., 2014; Mick and Pichora-Fuller, 2016; Contrera et al., 2017). It is possible that people with hearing loss withdraw from social situations due to difficulties in communicating and following conversations, which leads to social isolation. This association has been found more frequently in women compared to men (Mick et al., 2014), possibly due to several reasons: (i) Women feel more comfortable in reporting social isolation/loneliness compared to men (Shukla et al., 2020); (ii) women rely more on verbal communication for social networking (Maltz and Borker, 1982) and (iii) become socially and emotionally vulnerable if they are unable to effectively connect with their social network due to their hearing impairment (Shukla et al., 2020).

Hence, it is important to investigate the impact of untreated hearing loss on social and emotional loneliness, social isolation, perceived social support, and psychological discomfort (symptoms of depression, anxiety, stress).

## Materials and Methods

### Study Design and Setting

This was a cross-sectional study of a community-derived sample of volunteers and adults in contact with the clinical services of the Ear Science Institute of Australia in Perth, Western Australia. Participants were either native or fluent English speakers older than 40 years, did not use any hearing aids or hearing implants, had bilateral symmetrical pure tone audiometric thresholds of hearing sensitivity and did not have any morbidities or disabilities that prevented them from completing the assessments.

### Materials and Procedure

The assessment materials consisted of measures of hearing, mental health, social support and interaction, and social and emotional loneliness. The Human Research Ethics Committee of the University of Western Australia approved the study protocol (RA/4/1/7368), and all participants provided written informed consent.

#### Hearing Assessment

This included an otoscopic examination (OTOS/AA HDL otoscope, Welch Allyn, NY) and a pure-tone audiometric assessment (MIDIMATE 602 Audiometer, GN Otometrics Ltd., Sydney). Following an otoscopic examination, bilateral air conduction thresholds between 0.5 and 8 kHz and bone conduction thresholds between 0.5 and 4 kHz were obtained through standard audiometric assessment protocols conducted by a qualified audiologist in a standard sound-attenuated booth. These data were used to stratify participants into the following groups by their better ear four frequency pure-tone average thresholds at 0.5, 1, 2, and 4 kHz (BE4PTA): normal hearing (NH: 0–25 dB), mild to moderate sensorineural hearing loss (MMH: > 25–55 dB), moderately severe to profound sensorineural hearing loss (MSPH: 56+ dB).

#### Assessment of Social Isolation and Social Support

The Duke Social Support Index (DSSI-10) was used to measure the social interaction and satisfaction with social support received (Pachana et al., 2008; Wardian et al., 2013). The DSSI-10 contains two subscales of (a) social interaction (denoted SI) and (b) satisfaction with social support (denoted SS). The DSSI-10 has shown a good test-retest reliability, concurrent validity, and construct validity for the two independent subscales within the Australian Longitudinal Study on Women’s Health (Women’s Health Australia, 2004).

The social interaction subscale measures the size and structure of the social network and contains questions regarding the number of social interactions a person had within the past week with low social interaction subscale scores indicating greater social isolation (Freak-Poli et al., 2021b). The social support subscale measures perceived satisfaction with the behavioral or emotional support received from the social network and contained questions related to the subjective quality of those relationships with higher social support satisfaction subscale scores indicating better social support (Freak-Poli et al., 2021b).

#### Assessment of Loneliness

The de Jong-Gierveld loneliness scale (De Jong-Gierveld and Kamphuls, 1985) measured social and emotional loneliness. We used the short 6-item version of this scale (Gierveld et al., 2006), which contains three questions on social loneliness (SL) and three questions on emotional loneliness (EL). All statements are scored using a three-point scale (no, more or less, yes). To respond to the questions in this questionnaire, participants need to appraise their social relationships against their expectations (Valtorta et al., 2016). These are summed, with higher scores indicating higher levels of loneliness. Existing data support the validity and reliability of the scale (De Jong Gierveld and Van Tilburg, 2010).

#### Assessment of Psychological Discomfort (Depression, Anxiety, and Stress)

The Depression Anxiety Stress Scales: DASS-21 (Lovibond and Lovibond, 1995) was used to measure the severity (over the past 7 days) of a range of symptoms common to depression, anxiety, and stress. A 4-point combined severity/frequency scale is used to rate how the participant has experienced each question/statement over the past week. Each test item is scored from 0 (did not apply to me at all over the last week) to 3 (applied to me very much or most of the time over the past week). Seven statements are used to assess each of the three mental health domains, with total sub-scores for depression, anxiety, and stress calculated by summing the scores for the relevant items and multiplying them by two, so that each sub-score can range from 0 to 42 (Psychology Foundation of Australia, 2014).

#### Additional Measures

Participants self-reported their sex (male or female) and age (in years), years of formal education, physical exercises undertaken, smoking (current and past), alcohol consumption, and current living status (alone or with other people). We used the National Adult Reading Test-Revised (NART-R) to assess participants’ premorbid intellectual function scores (Nelson and Willison, 1991). All participants were asked to read aloud a list of 50 words from the NART-R test while the researcher recorded the number of errors made by the participant. The verbal intelligence quotient (VIQ) was calculated based on the NART-R error score.

### Statistical Analysis

The statistical analyses were carried out in SPSS Statistics for Windows version 25.0 (Armonk, NY: IBM Corp.) and R version 3.5.1 (R Core Team, 2018). Univariate linear regression was used to assess the association between Social Support, Social Interaction, and BE4PTA. The statically significant outcomes observed in the univariate analysis were then used in multiple linear regression. A *P*-value of less than 0.05 was considered to indicate statistical significance.

All authors accessed the study data and reviewed and approved the final manuscript. We used the STROBE statement to check the completeness of our study report (Von Elm et al., 2014).

## Results

Two hundred and two volunteers, aged between 40 and 88 years (*M* = 65.32 ± 11.07 years) took part in the study (Table 1). Of the 202 participants, 115 (57%) were male (*M* = 63.16 ± 12.05 years of age) and 87 (43%) were female (*M* = 68.17 ± 8.91 years of age). Detailed information on participant demographics is presented in Table 1.

**TABLE 1:** Demographic details of the participants.

Parameters	Total N(%)/Mean (*SD*)
**Gender**	
Female	115 (56.9)
Male	87 (43.1)
Age	65.32 (11.0)
NART-R	111.71 (6.6)
Depression	4.37 (5.0)
Anxiety	4.12 (4.5)
Stress	8.13 (6.2)
Emotional loneliness	0.76 (.9)
Social loneliness	0.95 (1.1)
Satisfaction with social support received	16.01 (2.3)
Social interaction	9.06 (2.0)
**Living alone**	
• Yes	41 (20.4)
• No	160 (79.6)
• Missing	1 (0.5)
**Exercise intensity**	
• Do not exercise	39 (19.3)
• Light	74 (36.6)
• Moderate	70 (34.7)
• High	14 (6.9)
• Missing	5 (2.5)
**Smoking**	
• No	125 (61.9)
• Current, everyday	11 (5.4)
• Current, social	1 (0.5)
• Used to smoke	58 (28.7)
• Missing	7 (3.5)
**Drinking**	
• Never	36 (17.8)
• Occasionally	120 (54.9)
• Frequently	42 (20.8)
• Missing	4 (2.0)
**BE4PTA categories**	
• No hearing loss	53 (26.2)
• Mild-moderate hearing loss	95 (47.0)
• Moderately severe to profound hearing loss	54 (26.7)
Musical training (mean years)	25.2 (139.3)

### Satisfaction With Social Support/Perceived Social Support

After univariate analysis, hearing was not statistically significant associated with SS. (*P* = 0.07). Poor satisfaction with social support scores was observed in individuals who live alone (*P* = 0.003), and experience more stress (*P* < 0.001), anxiety (*P* = 0.001), and depression (*P* < 0.001) or vice versa (see Tables 2, 3).

**TABLE 2:** Multi linear regression analysis results -better ear 4PTA average vs. social interaction and satisfaction with social support.

Variables	SE	*R* ^2^	Beta (Stand)	OR (95%CI)	*P*
Social interaction (SI)	0.9	0.0	−0.1	−0.1 (−3.4 to 0.4)	0.1
Satisfaction with social support (SS)	0.8	0.0	−0.124	−0.1 (−3.1 to 0.2)	0.1

**TABLE 3:** Univariate and multivariate analysis, satisfaction with social support received (SS).

	Univariate					Multivariate				
Variable	B (95%CI)	SE	*R* ^2^	Beta (Stand)	*P*	B (95%CI)	SE	Beta (Stand)	*R*^2^ 0.161	*P*
Gender	−0.04 (−1.1 to 0.16)	0.33	0.01	−0.10	0.14					
Age	0.01 (−0.02 to 0.03)	0.01	0.00	0.04	0.52					
NART-R	0.02 (−0.02 to 0.07)	0.02	0.00	0.06	0.34					
Education	0.014 (−0.101 to 0.128)	0.06	0.00	0.24	0.81					
Drinking Never	REF									
Occasionally	0.60 (−0.23 to 1.44)	0.42		0.12	0.15					
Frequently	0.47 (−0.55 to 1.50)	0.51		0.08	0.36					
Smoking			0.01							
Non-smoker	REF									
Current everyday	0.83 (−0.61 to 2.28)	0.73		0.08	0.25					
Current-social	1.01 (−3.61 to 5.64)	2.34		0.03	0.66					
Used to smoke past	−0.01 (−0.85 to 0.65)	0.37		−0.02	0.79					
Exercise intensity	REF	0.45	0.01	0.14	0.13					
Do not exercise	0.68 (−0.20 to 1.55)	0.45		0.15	0.11					
Light	0.73 (−0.15 to 1.61)	0.71		0.01	0.84					
Moderate	0.14 (−1.27 to 1.55)									
High										
Years musical experience	0.01 (−0.01 to 0.04)	0.01	0.01	0.07	0.32					
**Living arrangement**	−**1.20 (**−**2.00 to**−**0.42)**	**0.40**	**0.04**	−**0.20**	0.003*	−**1.04 (**−**1.79 to**−**0.29)**	**0.38**	−**0.18**		0.006*
**Stress**	−**0.084 (**−**0.13 to**−**0.03)**	**0.02**	**0.05**	−0**.22**	0.001*	−0.01 (−0.07 to 0.05)	0.03	−0.03		0.70
**Anxiety**	−**0.12 (**−**0.19 to**−**0.05)**	**0.03**	**0.05**	−0**.23**	0.001*	−0.01 (−0.09 to 0.08)	0.04	−0.01		0.90
**Depression**	−**0.17 (**−**0.23 to**−**0.11)**	**0.03**	**0.13**	−0**.36**	0.000*	−**0.16 (**−**0.25 to**−**0.080)**	**0.04**	−**0.34**		0.000*
Better ear 4pta average	−0.01 (−0.02 to 0.01)	0.01	0.01	−0.12	0.07					
Depression*hearing	0.000 (−0.00 to 0.00)	0.00	0.13	−0.02	−0.88					
Stress*hearing	0.000 (−0.00 to 0.00)	0.00	0.06	−0.06	0.71					
Anxiety*hearing	−0.00 (−0.00 to 0.00)	0.00	0.06	−0.10	0.53					
Living*hearing	0.01 (−0.01 to 0.04)	0.01	0.06	0.11	0.87					

*P < 0.05.

### Social Interaction/Social Isolation

Two variables have a significant effect on SI in the univariate analyses: depression (*P* = 0.01) and age (*P* < 0.001). After multivariate analysis for confounders, aging (*P* = 0.01) and depression (*P* = 0.01) continued to be statistically significant. These results suggest that higher depression scores and older age contributes to increased social isolation. A statistically significant interaction between age and hearing was observed (*P* = 0.04). Poorer SI scores are seen for people who are not only older but with poorer hearing. Results are summarized in Table 4. No significant effect of years of formal education, premorbid IQ (NART-R), smoking, alcohol ≥ 14 drinks per week or exercise were observed on SI and SS.

**TABLE 4:** Univariate and multivariate analyses, social interaction (SI).

Variable	Univariate					Multivariate				
	B (95%CI)	SE	*R* ^2^	Beta (stand)	P	B (95%CI)	SE	Beta (stand)	R^2^	P
Gender	−0.21 (−0.79 to 0.36)	0.19	0.02	−0.05	0.46	−				
Age	**0.03 (0.01 to 0.06)**	**0.01**	**0.03**	**0.18**	0.00*	**0.03 (0.00 to 0.06)**	**0.02**	**0.18**	**0.06**	0.00*
Education	0.29 (−0.07 to 0.13)	0.05	0.00	0.56	0.57					
NART-R	0.03 (−0.00 to 0.07)	0.02	0.01	0.11	0.10					
Drinking			0.00							
Never	REF	0.37		0.04	0.65					
Occasionally	0.16 (−0.57 to 0.90)			0.11	0.21					
Frequently	0.56 (−0.33 to 1.46)	0.45								
Smoking			0.00							
Non-smoker	REF									
Current everyday	−0.58 (−1.86 to 0.68)	0.64		−0.06	0.36					
Current-social	1.86 (−2.21 to 5.95)	2.07		0.06	0.36					
Used to smoke	−0.17 (−0.82 to 0.48)	0.33		−0.03	0.61					
Exercise intensity			0.01							
Do not exercise	REF									
Light	0.45 (−0.32 to 1.22)	0.39		0.10	0.25					
Moderate	0.40 (−0.37 to 1.18)	0.39		0.09	0.30					
High	−0.51 (−1.75 to 0.73)	0.63		−0.06	0.42					
Years musical experience	0.00 (−0.21 to 0.02)	0.01	0.00	0.01	0.83					
Living arrangement	−0.10 (−0.82 to 0.60)	0.36	0.00	−0.02	0.77					
Stress	−0.041 (−0.06 to 0.00)	0.02	0.01	−0.12	0.08					
Anxiety	−0.004 (−0.06 to 0.05)	0.03	0.00	−0.00	0.89					
**Depression**	−**0.07 (**−**0.12 to**−**0.01)**	**0.02**	**0.02**	−**0.17**	0.01*	**0.06 (**−**0.12 to**−**0.01)**	**0.02**	**−0.16**	**0.05**	0.01*
Better ear 4pta average	−0.008 (−0.01 to 0.00)	0.00	0.01	0.11	0.11					
Depression*hearing	0.000 (0.00 to 0.00)	0.00	0.03	−0.05	0.66					
Age*hearing	−**0.001 (**−**0.002 to 0.00)**	**0.00**	**006**	−**0.75**	0.04*					

*P < 0.05.

### Emotional Loneliness

The total number and percentage of participants for each EL and SL category are reported in Table 5. Logistic multinominal regression analysis did not show any effect of gender, premorbid IQ (NART-R), alcohol ≥ 14 drinks per week, smoking and exercises on EL. Living alone (*P* = 0.01) significantly contributed to the intense levels of emotional loneliness. Age (*P* = 0.02) and (*P* = 0.02), depression (*P* < 0.001) and (*P* < 0.001), anxiety (*P* < 0.001) and (*P* < 0.001), stress (*P* < 0.001) and (*P* < 0.001), BE 4PTA (*P* < 0.001) and (*P* < 0.001) significantly contributed to both moderate and intense levels of EL, respectively (see Tables 5, 6).

**TABLE 5:** Nominal regression emotional loneliness (EL) and social loneliness (SL).

Variable	Category	Category explanation	Number of participants in each category	Percentage of participants in each category
Emotional loneliness (EL)	0	Not emotionally lonely	104	51.5%
	1	Mildly emotionally lonely	58	28.7%
	2	Moderately emotionally lonely	25	12.4%
	3	Intensely emotionally lonely	15	7.4%
Social loneliness (SL)	0	Not socially lonely	104	52.5%
	1	Mildly socially lonely	29	14.6%
	2	Moderately socially lonely	34	17.2%
	3	Intensely socially lonely	31	15.7%

**TABLE 6:** Association between Age, living arrangement, depression, anxiety, stress, and better ear 4PTA hearing thresholds on EL categories.

Variable	EL Category	B (95% CI)	SE	Exp (B)	P
Age	1	−0.01 (0.95 to 1.01)	0.01	0.98	0.30
	**2**	−**0.04 (0.92 to 0.99)**	**0.02**	**0.95**	0.02*
	**3**	−**0.05 (0.90 to 0.99)**	**0.02**	**0.95**	0.02*
Gender	1	−0.42 (0.34 to 1.26)	0.33	0.65	0.20
	2	−0.75 (0.18 to 1.18)	0.47	0.47	0.11
	3	−1.01(0.10 to 1.21)	0.61	0.36	0.10
Education	1	−0.04 (0.90 to 1.13)	0.02	1.01	0.92
	2	−0.06 (0.79 to 1.07)	0.03	0.92	0.27
	3	−0.06 (0.84 to 1.23)	0.04	1.01	0.90
NART-R	1	−0.04 (0.91 to 1.10)	0.02	0.96	0.11
	2	−0.06 (0.87 to 0.99)	0.03	0.93	0.05
	3	−0.06 (0.87 to 1.01)	0.04	0.94	0.12
Living arrangement	1	0.22 (0.55 to 2.81)	0.41	1.24	0.60
	2	−0.04 (0.30 to 3.13)	0.60	0.95	1.00
	**3**	**1.43 (1.34 to 13.00)**	**0.60**	**4.20**	0.01*
Depression	1	0.08 (1.00 to 1.20)	0.04	1.10	0.90
	**2**	**0.31 (1.22 to 1.53)**	**0.05**	**1.40**	0.00*
	**3**	**0.29 (1.20 to 1.50)**	**0.61**	**1.33**	0.00*
Anxiety	1	0.00 (0.90 to 1.09)	0.04	1.00	1.00
	**2**	**0.20 (1.10 to 1.33)**	**0.04**	**1.21**	0.00*
	**3**	**0.20 (1.09 to 1.40)**	**0.05**	**1.22**	0.00*
Stress	1	0.04 (1.00 to 1.11)	0.03	1.05	0.10
	**2**	**0.16 (1.19 to 1.30)**	**0.03**	**1.18**	0.00*
	**3**	**0.16 (1.08 to 1.29)**	**0.04**	**1.18**	0.00*
BE4PTA	1	0.04 (0.99 to 1.01)	0.00	1.00	0.47
	**2**	**0.01 (0.99 to 1.02)**	**0.00**	**1.01**	0.00*
	**3**	**0.02 (1.00 to 1.04)**	**0.09**	**1.02**	0.00*
Musical experience (Ref yes)	1	0.00 (0.98 to 1.03)	0.01	1.00	0.70
	2	0.02 (0.93 to 1.02)	0.02	0.98	0.39
	3	−0.04 (0.95 to 1.04)	0.02	1.00	0.86
Smoking (Ref yes)	1	0.17 (0.60 to 2.34)	0.34	1.19	0.60
	2	−0.27 (0.31 to 1.86)	0.45	0.76	0.55
	3	1.43 (0.89 to 19.57)	0.78	4.19	0.06
Drinking (Ref yes	1	−0.20 (0.34 to 1.95)	0.44	0.81	0.65
	2	0.58 (0.65 to 1.94)	0.51	1.79	0.25
	3	−1.16 (0.03 to 2.52)	1.06	0.31	0.27
Exercise (Ref yes)	1	−0.38 (0.30 to 1.55)	0.41	0.68	0.36
	2	0.65 (0.74 to 4.98)	0.48	1.92	0.17
	3	−0.22 (0.20 to 3.08)	0.68	0.80	0.74

*P < 0.05.

An adjacent category ordinal regression analysis was conducted to investigate this association between EL and BE 4PTA, living alone and age. The adjacent category logistic regression model was implemented in the VGAM framework (Yee and Wild, 1996; Yee, 2010, 2015) using the R statistical language (R Core Team, 2018). This adjacent ordinal model considers the impact of the predictors on the odds ratio on the adjacent ordinal response levels with the following mathematical model,

$$L_{i}=\log_{e}\left(\frac{p_{i+1}}{p_{i}}\right)=X_{\text{i}}\beta_{\text{i}},\mathrm{where}p_{i}=P\left(EL=i\right).$$

In our case, the *p*_*i*_ denote the probabilities of being at an emotional loneliness level *i*. This is modeling the log-odds as a linear function of the predictors in design matrix *X* and the linear predictor β. Therefore, the full model would involve the three logistic equations,

$$\begin{array}{cc} L_{0} & ={\text{log}}_{e}\,\left(\frac{p_{1}}{p_{0}}\right)={\text{log}}_{e}\,\left(\frac{P\left(EL=1\right)}{P\left(EL=0\right)}\right)=X\,\beta_{1}\\ L_{1} & ={\text{log}}_{e}\,\left(\frac{p_{2}}{p_{1}}\right)={\text{log}}_{e}\,\left(\frac{P\left(EL=2\right)}{P\left(EL=1\right)}\right)=X\,\beta_{2}\\ L_{2} & ={\text{log}}_{e}\,\left(\frac{p_{3}}{p_{2}}\right)={\text{log}}_{e}\,\left(\frac{P\left(EL=3\right)}{P\left(EL=2\right)}\right)=X\,\beta_{3}. \end{array}$$

Only three equations are modeled since there are only three transitions to cover the four EL ordinal levels. The likelihood ratio test was used to investigate the proportional odds assumption, i.e., if there are different predictor effects at different ordinal levels, but there was no statistical evidence for this with a *p*-value of 0.86, the same effect of the predictors is present across all adjacent transitions; only an intercept adjustment is required. Furthermore, there was no statistical evidence to suggest an interaction between the predictor terms. The final model is,

$$\begin{array}{c} \frac{P\left(EL=i+1\right)}{P\left(EL=i\right)}={\text{e}}^{\beta_{0},i}\cdot {\left(1.546\right)}^{L\,i\,v\,i\,n\,g\,S\,t\,a\,t\,u\,s}\cdot {\left(0.9754\right)}^{A\,g\,e}\\ \cdot {\left(1.114\right)}^{D\,e\,p}\,\cdot {\left(1.006\right)}^{B\,E\,4\,P\,T\,A}, \end{array}$$

where β_0,*i*_ is an intercept term for each transition level that gives the estimated log odds for the adjacent levels at the baseline predictor level. Here, the baseline predictors are for participants that live alone (Living alone 0). The living alone variables in the equation above are indicators that take the value 1 for participants that live alone. The exponentiated effect estimates are shown above in the equation. The corresponding *p*-values and 95% CI are BE4PTA [0.048, (95% CI: 1.00 to −1.01)], Age [0.002, (95% CI: 0.95 to −0.99)], Living alone [0.044, (95% CI: 1.01 to −2.36)], and Depression [2.5×10^−9^, (95% CI: 1.06–1.16)]. Interpreting this model suggests that a participant living alone increases their odds of transitioning to the next higher EL level by 54.6%. For each unit of BE4PTA, a participant’s odds of transitioning to the next higher EL level increased by 0.6%. For example, if a person whose hearing deteriorates from no hearing loss to mild to moderate hearing loss, where the BE4PTA increases from 25 to 50 dB, they will have 1.006^25^ = 1.16 or a 16% increase in their odds of transitioning to the next higher EL level.

### Social Loneliness

Multinominal analysis revealed that age, gender, premorbid IQ (NART-R), anxiety, BE4PTA, smoking, and alcohol ≥ 14 drinks per week had no effect on SL. Three of the variables exhibited a statistically significant association with the SL response. Living alone (*P* = 0.01), depression (*P* = 0.01), and stress (*P* = 0.01) had a significant impact on intense levels of SL. Results are summarized in Table 7.

**TABLE 7:** Association between living arrangement, depression, and Stress on SL categories.

Variable	EL category	B (95%CI)	Std.Error	Exp (B)	Sig
					
Age	1	−0.02 (0.93 to 1.00)	0.01	0.97	0.13
	2	−0.02 (0.94 to 1.01)	0.01	0.97	0.19
	3	−0.03 (0.93 to 1.00)	0.01	0.97	0.09
Gender	1	0.14 (0.44 to 2.29)	0.41	1.01	0.97
	2	−0.19 (0.37 to 1.81)	0.40	0.82	0.62
	3	0.21 (0.55 to 2.77)	0.40	1.24	0.59
Education	**1**	**0.25 (1.09 to 1.51)**	**0.08**	**1.28**	0.002*
	**2**	**0.16 (1.01 to 1.35)**	**0.07**	**1.17**	0.031*
	3	0.12 (0.98 to 1.31)	0.07	1.13	0.09
NART-R	1	−0.00 (0.94 to 1.06)	0.03	0.99	0.97
	2	0.02 (0.96 to 1.09)	0.03	1.02	0.36
	3	0.03 (0.96 to 1.10)	0.03	1.03	0.32
Living alone	1	−0.21 (0.24 to 2.60)	0.60	0.80	0.71
	2	0.60 (0.70 to 4.77)	0.48	1.82	0.22
	**3**	**1.04 (1.29 to 6.84)**	**0.44**	**2.88**	0.01*
Depression	1	0.03 (0.10 to 1.13)	0.04	1.03	0.43
	2	0.05 (0.10 to 1.14)	0.04	1.05	0.17
	**3**	**0.12 (1.05 to 1.21)**	**0.04**	**1.13**	0.00*
Anxiety	1	0.01 (0.92 to 1.14)	0.04	1.01	0.71
	2	0.05 (0.97 to 1.14)	0.04	1.05	0.22
	3	0.04 (0.96 to 1.14)	0.04	1.04	0.28
Stress	1	0.07 (1.00 to 1.15)	0.03	1.07	0.08
	2	0.05 (0.99 to 1.13)	0.03	1.06	0.07
	**3**	**0.10 (1.03 to 1.27)**	**0.03**	**1.10**	0.00*
BE 4PTA	1	0.00 (0.98 to 1.01)	0.00	1.00	0.65
	2	−0.00 (0.98 to 1.01)	0.00	0.99	0.63
	3	0.00 (0.99 to 1.01)	0.00	1.00	0.55
Musical experience (Ref yes)	1	−0.04 (0.90 to 1.01)	0.03	0.95	0.17
	2	0.10 (0.98 to 1.04)	0.01	1.01	0.29
	3	−0.02 (0.92 to 1.02)	0.02	0.97	0.25
Smoking (Ref yes)	1	0.35 (0.63 to 3.22)	0.41	1.43	0.38
	2	0.44 (0.55 to 4.43)	0.53	1.55	0.40
	3	0.54 (0.62 to 4.72)	0.51	1.72	0.29
Drinking (Ref yes	1	−0.44 (0.25 to 1.65)	0.48	0.64	0.36
	2	−0.77 (0.12 to 1.73)	0.67	0.46	0.25
	3	−0.70 (0.14 to 1.72)	0.63	0.49	0.26
Exercise (Ref yes)	1	0.82 (0.72 to 7.12)	0.58	2.27	0.15
	2	0.03 (0.23 to 4.61)	0.76	1.04	0.95
	3	1.13 (0.86 to 11.12)	0.65	3.10	0.08

Reference SL: 0. *P < 0.05.

## Discussion

This study investigated the association between untreated hearing loss and three aspects of social health: social isolation, loneliness, and social support in older adults and psychological discomfort.

The key finding is that untreated hearing loss was not associated with social loneliness but was significantly associated with emotional loneliness. For every unit of increase in hearing loss, the odds of transitioning from mild to moderate or moderate to intense levels of emotional loneliness increased by 0.6%. If an older adult’s hearing deteriorates from mild-moderate, the chances of moving from mild-moderate or moderate to intense levels of emotional loneliness are 16%.

Weiss (1973) differentiated between emotional and social loneliness (Weiss, 1973). Emotional loneliness results from an absence of an intimate or a close emotional relationship (a partner, a best friend), and it is associated with feelings of abandonment, aloneness, and anxiety. Social loneliness results from the lack of many contacts or social networks (friends, colleagues, neighbors) (Weiss, 1973). Loneliness is more closely related to the quality than quantity of social interactions (Hawkley et al., 2008). Communication is a two-way process involving both the person with a hearing loss and their communication partner (Scarinci et al., 2008). Intimate, close partner relationships can suffer considerably from hearing loss (Scarinci et al., 2008). Studies on challenges experienced by hearing-impaired people and their significant others have reported difficulties communicating in background noise, annoyance due to having to repeat during a conversation, frustration and/or annoyance due to hearing loss (Stark and Hickson, 2004). Based on our data, we posit that the breakdown in communication resulting from moderate to severe levels of hearing impairment could have a significant impact on close relationships resulting in emotional loneliness.

Particularly noteworthy are the findings that living alone increases the odds of transitioning from mild-moderate or moderate-intense levels of emotional loneliness by 54.6%. This is a significant concern as older adults who live alone and experience emotional loneliness seem to be at greater risk of all-cause mortality (HR = 1.186, *p* = 0.029, 95% CI = 1.017–1.383) compared to older adults who live with someone else and experience emotional loneliness (O’Súilleabháin et al., 2019). We posit that, even if older adults live with someone, if their intimate or close relationship is compromised due to a communication barrier due to untreated hearing loss, they could be at greater risk of all-cause mortality than normal hearing- or hearing-impaired older adults using hearing rehabilitation devices.

We also found that those with high emotional and social loneliness scores also had high depression and stress scores and were living alone. Our findings complement those of Alpass and Neville (2003), who found a significant association between loneliness and depressive symptoms in 217 older men. Similarly, Savikko et al. (2005) reported that living alone or in a residential home is a risk factor for loneliness. In contrast, poor health and functionality, vision and hearing impairment increases the prevalence of risk of loneliness.

The theory of conservation resources states that wellbeing can be social or psychological (Hobfoll, 1989, 2001). Losses in psychological resources such as mental health increase the likelihood of loneliness (Aartsen and Jylhä, 2011). Further, reductions in personal resources, compared to social resources, strongly predict loneliness (Fry and Debats, 2002). Our previously published results reported a significant association between clinically significant depression, stress and anxiety and untreated hearing loss (Jayakody et al., 2018). A significant association between loneliness and increases in depressive symptoms (Cacioppo et al., 2006, 2010; Sung et al., 2016) has a reciprocal influence on loneliness and depressive symptoms over time (Cacioppo et al., 2006).

We also explored the association between untreated hearing loss and social isolation (through social interaction) and perceived social support (satisfaction with social support received). Those who had higher depression scores showed increased social isolation scores and poor social support. Previous studies have shown that social support and depression share a bidirectional relationship (Stice et al., 2004; Almquist et al., 2016). According to the stress-buffer mechanism, social support attenuates the effects of stressful life events through effective coping strategies (Cohen and Wills, 1985). It influences positive health-related behaviors by regulating emotional responses to stressful or other high-risk situations (Cohen, 1988, 2004).

We failed to observe any significant association between untreated hearing loss and social isolation or perceived social support. The underlying reasons for these results are unclear. DSSI-10 measures both structural (number, diversity, density of a person’s social network) and functional aspects (involvement, perceived availability, and adequacy) of social relationships (Valtorta et al., 2016); however, it does not explicitly measure the impact of communication impairment on social interaction/isolation or perceived social support. Future studies should investigate this association using tools that are more sensitive to the effects of communication impairment resulting from hearing loss.

On the other hand, we found that interaction between aging and hearing loss significantly contributed to social interaction/isolation. Aging reduces the number of social contacts and brings a decline in social activity (Dykstra et al., 2005). Epidemiological data also document an increase in hearing loss due to aging (Cruickshanks et al., 2003; Gopinath et al., 2009). Hearing loss imposes more demands on communicating partners, such as speaking slowly, exaggerating articulatory movements, and moving closer to the listener (Arlinger, 2003). These increases in demands may make people less in contact with hearing-impaired individuals (Arlinger, 2003). Hence, it is no surprise that aging and age-related hearing loss significantly impairs social relationships by reducing social networks’ size and structure.

In summary, we observed a significant relationship between untreated hearing loss and emotional loneliness. Depression was a common factor that contributed to loneliness, social isolation, and poor social support. West (2017) reported that social support moderates the association between self-reported hearing loss and depression. Excluding the additional costs associated with using the health care systems, such as more general practitioner visits, medications, emergency services, outpatient and hospital admissions due to social isolation/loneliness (Longman et al., 2013), the cost of social isolation and loneliness to the Australian economy is AUD$1.7 billion (Australian Institute of Health and Welfare [AIHW], 2021). Some of these costs are due to the consequences associated with social isolation/loneliness; however, some are due to lonely people visiting physicians to satisfy their need for social relationships (Gerst-Emerson and Jayawardhana, 2015). Similarly, in audiological clinical practice, we often come across older adults coming in for hearing aid programming or cochlear implant mapping appointments to compensate for their loneliness.

The following steps can be taken to increase the social support and help reduce the burden of loneliness and isolation: (i) encourage the use of engaged coping strategies such as managing the hearing loss using hearables/hearing aids or hearing implants or using communication strategies (Heffernan et al., 2016); (ii) provide training to communication partners/significant others on support socially the person with hearing impairment (Preminger and Meeks, 2010) and (iii) train audiologists in providing counseling and emotional support to their patients (Saunders et al., 2021).

### Limitations

This is a cross-sectional study; therefore, results must be interpreted cautiously. A study with more power and a longitudinal design could provide more insight into the causal relationship between untreated hearing loss, loneliness, social isolation, and social support.

### Future Directions

A follow-up manuscript will investigate the effect of hearing loss treatment using hearing aids/hearing implants that could help alleviate loneliness, social isolation, and depression. Future research is required to investigate whether social support could buffer the association between hearing loss and emotional loneliness.

## Data Availability Statement

The raw data supporting the conclusions of this article will be made available by the authors, without undue reservation.

## Ethics Statement

The studies involving human participants were reviewed and approved by the Human Research Ethics Committee of the University of Western Australia approved the study protocol (RA/4/1/7368). The patients/participants provided their written informed consent to participate in this study.

## Author Contributions

TM, JY, and JG-S contributed to the data collection. DJ, OA, and RE contributed to the study design, interpretation of results, and manuscript writing. JW and IS contributed to the analysis, interpretation of data, and drafting the manuscript. All authors contributed to the article and approved the submitted version.

## Conflict of Interest

The authors declare that the research was conducted in the absence of any commercial or financial relationships that could be construed as a potential conflict of interest.

## Publisher’s Note

All claims expressed in this article are solely those of the authors and do not necessarily represent those of their affiliated organizations, or those of the publisher, the editors and the reviewers. Any product that may be evaluated in this article, or claim that may be made by its manufacturer, is not guaranteed or endorsed by the publisher.
